# Health care workers indicate ill preparedness for Ebola Virus Disease outbreak in Ashanti Region of Ghana

**DOI:** 10.1186/s12889-017-4474-6

**Published:** 2017-06-06

**Authors:** Augustina Angelina Annan, Denis Dekugmen Yar, Michael Owusu, Eno Akua Biney, Paa Kobina Forson, Portia Boakye Okyere, Akosua Adumea Gyimah, Ellis Owusu-Dabo

**Affiliations:** 1 Kumasi Centre for Collaborative Research in Tropical Medicine, Kwame Nkrumah University of Science and Technology, Kumasi, Ghana; 2 Komfo Anokye Teaching Hospital, Kumasi, Ghana; 3 Kumasi South Hospital, Ghana Health Services, Kumasi, Ghana; 4 School of Public Health, Kwame Nkrumah University of Science and Technology, Kumasi, Ghana; 5 Department of Theoretical and Applied Biology, Kwame Nkrumah University of Science and Technology, Kumasi, Ghana

**Keywords:** Ebola Virus disease, Healthcare workers, Preparedness, Ghana

## Abstract

**Background:**

The recent Ebola Virus Disease (EVD) epidemic that hit some countries in West Africa underscores the need to train front line high-risk health workers on disease prevention skills. Although Ghana did not record (and is yet to) any case, and several health workers have received numerous training schemes, there is no record of any study that assessed preparedness of healthcare workers (HCWS) regarding EVD and any emergency prone disease in Ghana. We therefore conducted a hospital based cross sectional study involving 101 HCWs from two facilities in Kumasi, Ghana to assess the level of preparedness of HCWs to respond to any possible EVD.

**Methods:**

We administered a face-to-face questionnaire using an adapted WHO (2015) and CDC (2014) Checklist for Ebola Preparedness and assessed overall knowledge gaps, and preparedness of the Ghanaian HCWs in selected health facilities of the Ashanti Region of Ghana from October to December 2015.

**Results:**

A total 92 (91.09%) HCWs indicated they were not adequately trained to handle an EVD suspected case. Only 25.74% (*n* = 26) considered their facilities sufficiently equipped to handle and manage EVD patients. When asked which disinfectant to use after attending to and caring for a suspected patient with EVD, only 8.91% (*n* = 9) could correctly identify the right disinfectant (χ^2^ = 28.52, *p* = 0.001).

**Conclusion:**

Our study demonstrates poor knowledge and ill preparedness and unwillingness of many HCWs to attend to EVD. Beyond knowledge acquisition, there is the need for more training from time to time to fully prepare HCWs to handle any possible EVD case.

## Background

During the last outbreak of Ebola Virus Disease (EVD) and its consequential massive epidemic with very high mortality [1], many health systems and services in West Africa were overwhelmed and disrupted. This was partly due to the poor and weak health systems coupled with unprepared and unskilled frontline healthcare workers (HCWs) compounded by poor understanding of the disease dynamics tied to lack of requisite resources. During the early part of 2014, the emergence of EVD [1] in Guinea, jolted the health care systems of West African sub-region claiming over 9800 lives [2] including more than 491 (58.7%) deaths of HCWs from 839 infections [2]. This epidemic therefore reinforced the fact that HCWs are at high-risk of being infected with the disease in line with their core duties. Empirical data generated during and after the epidemic indicated how unprepared most HCWs were in the face of the crisis. Studies in Nigeria, Guinea and India indicate the low level of knowledge, negative attitude and sub-standard practices that can be eliminated through continued training of HCWs as well as provision of needed and adequate resources in their line of duties [3–6].

The countries worst hit were Liberia, Sierra Leone, Guinea and several other countries with imported cases [7]. Like most West African nations, Ghana was on high alert and was number one on the list of countries deemed to be at high risk of EVD. Thus, the country tried to make some preparations in the wake of the epidemic [8]. The government with support from donor organizations such as the World Health Organization (WHO), Médecins sans frontières (MSF) put in place resources for training of health professionals and some level of retooling of hospitals and preparedness of health workers in the face of any possible emergency scenarios. Various HCWs received both theoretical and practical training on how to manage infected and affected persons. These training sessions took the form of onsite and off site coaching as well as workshops. Simulation exercises were also conducted to bring to bear how HCWs would react during EVD emergency scenarios. For example, the German government through the Kumasi Centre for Collaborative Research in Tropical Medicine organized hands on training for several West African nationals on sample taking, sample testing and donning and doffing personal protective equipment (http://kccr.org). More importantly, there was the construction of three treatment centres and as well, a standby ambulance service for transportation of confirmed cases to the treatment centres. Incidentally, Ghana did not record any case in the wake of the epidemic and has so far not recorded any case of EVD. However, the response of HCWs to the scenarios identified several gaps. Following a series of training for HCWs, one could easily assume that health care workers are adequately prepared and equipped with the requisite knowledge and skills to deal with any possible EVD outbreak. It is unclear for example to what extent these exercises were practiced and for how long they were made a part of routine hospital activities. One therefore wonders how well prepared HCWs within Ghana are to responding not only to EVD but other epidemic prone diseases (EPDs) requiring a concerted approach to preparedness and management.

Even though some resources have been invested in response to the EVD scare in Ghana, there has not been any assessment on the preparedness of health workers in the face of any possible emergency scenarios. Simply providing the tools such as medications, personnel protective equipment (PPE) and other logistics is no panacea for adequately and appropriately responding to EPDs. Consequently, if healthcare staff lack the basic knowledge, practical and organizational skills to plan and respond to such emergency situations, it would not only spell doom for themselves but also for the general population as was the case of the recent epidemic in West Africa. It is important for example to understand the dynamics of what will propel a HCW to be willing to put his or her life in the line of duty for a case of EVD. It is therefore critical to understand current preparedness of the healthcare worker in Ghana in order to make recommendations for future training and planning for any epidemics situation. The need for Ghana to therefore have empirical data on general emergency preparedness to determine and understand knowledge gaps, and to assess knowledge versus practice in a bid to provide direction for policy cannot be overemphasized. In view of this, we therefore assessed the level of preparedness, readiness and knowledge of EVD emergency response among a population of healthcare workers (HCWs) in the Kumasi Metropolis of Ashanti Region, Ghana.

## Methods

### Study design and setting

We conducted a hospital based cross-sectional study among healthcare workers at the Kumasi South and Suntreso Government Hospitals designated as “advanced Ebola holding unit” and “Ebola standing team” respectively, in the Kumasi Metropolis. The Kumasi South and Suntreso hospitals have an average monthly Out Patient Department (OPD) attendance of about 20,603 and 11,712 patients respectively and health staff of approximately 450 each. Similar to most facilities, there are more females nurses than males.

We organized a day’s training for our research assistants on how to use Personal Digital Assistant device (PDAs) Samsung Galaxy note 8 GT-N5100 (Samsung Electronics Co. Ltd., Seoul, Korea) in capturing data.

The original version of the questionnaire was pretested, with five healthcare workers who were similar in their characteristics to the members of the study population but outside the area of jurisdiction and study to ensure validity and measurement bias. The questionnaire was revised based on the suggestions and comments (mainly on how the questions had been constructed) obtained from the pilot. This was the final and validated data capturing tool used during the study.

At both facilities, we contacted the Medical Superintendents to obtain permission to attend their morning meetings to explain the aims and objectives of the work to HCWs. During this time, HCWs were given the opportunity to ask questions. Two field assistants were stationed at each of the study sites for data capture. Some of the questions asked included the organism responsible for EVD, the mode of transmission of the disease, HCW preparedness to handle any EVD case and among other things early clinical features of the infection.

### Data collection

In estimating the sample size for this study, previous data from the hospital indicates that there are approximately 900 HCWs at the two facilities. Assuming a 95% confidence interval and if 70% of these HCWs would come into contact with an EVD suspected case, allowing an error rate of 10%, approximately 87 HCWs would provide a default study power of 80% and an alpha of 5%. With approximately a non-response rate of 15% allowing us to sample 101 HCWs from the two facilities providing emergency services within the Ashanti Region of Ghana.

Any healthcare worker attending directly to patients in emergency situation was therefore eligible for inclusion in the study. Our sampling frame consisted of a list of a total of 200. From this list, we then took a systematic random sample of all eligible health workers to represent the sample size. After obtaining written informed consent indicated by signature and or thumbprint of participants, we then administered the questionnaires within the two facilities.

We used the WHO (2015) and CDC (2014) Checklist for Ebola Preparedness that provides practical and specific suggestions to ensure that health facilities are able to help their personnel detect possible Ebola cases, protect personnel, and respond appropriately [9, 10]. This checklist included facility evaluation, knowledge and preparedness of HCWs. Based on these checklists we developed a questionnaire to ascertain the overall knowledge and preparedness of Ghanaian HCWs on EVD outbreak. Our questionnaire was administered from a PDA and recorded each participant’s demographics, preparedness, form of compensation HCWs think would be appropriate when taking care of EVD case, and knowledge of EVD during the period October to December 2015. Answers to these questions were needed from HCWs to determine information access on EVD among HCWs, their knowledge about EVD and the form of compensation HCWs think would be appropriate when taking care of EVD case among others.

### Data management

Data were collected electronically using tablets for cloud storage through CommCare ODK version 2.27.2, aggregated into Microsoft Excel file, exported into STATA version 14 and analyzed. Descriptive statistics was used to summarize the distribution of various variables into tables and figures. Categorical variables were analyzed using chi-square tests and logistic regression for associations.

## Results

### Background of the study participants

Table 1 shows the background characteristics of the study participants. A total of 101 study participants were interviewed, of which 85 (84.16%) were females. Respondents were categorized into three main groups by occupation: Nurses (76.24%), Medical Doctors (19.80%) and Physician Assistants (PA) (3.96%). Majority (54.46%) of the respondents were married. A total 52.48% (53) had been practicing their profession for less than 5 years (SD = 9.22 ± 10.52 years). At both facilities, 75.25% (76) of the respondents had been working in the facility for less than 5 years (SD = 4.04 ± 4.07 years).

**Table 1:** Demographic Characteristics of participating Healthcare Worker (HCWs)

Variable	Frequency	(%)
Gender		
Male	16	15.84
Female	85	84.16
Profession		
Medical Doctor	20	19.80
Physician Assistant	4	3.96
Nurse	77	76.24
Marital status		
Married	55	54.46
Divorced/Widowed	3	2.98
Single	43	42.57
Tenure (Years)		
≤ 5	53	52.48
6–10	20	19.80
≥ 11	28	27.27
Mean ± SD	9.22 ± 10.52	1–19
Years in current health facility		
≤ 5	76	75.25
6–10	17	16.83
≥ 10 y	8	7.92
Mean ± SD	4.04 ± 4.07	1–19

### Knowledge and awareness of EVD

Table 2 shows the participants knowledge and awareness of EVD. Of the 101 HCWs interviewed, 83.17% (*n* = 84) correctly identified the cause of EVD, 13.86% (*n* = 14) did not know the cause, while 2.97% (*n* = 3) incorrectly labeled the cause to be a bacterium. Even though one (0.99%) Doctor and 16 (15.84%) Nurses were unable to correctly identify the cause; no group was significantly likely to incorrectly label the cause of EVD (χ^2^ = 5.41, *p* = 0.247).

**Table 2:** Knowledge and awareness of Ebola Virus Disease (EVD) by HCW category

Question	HCW category Total, (%)				
	MDs (*n* = 20)	Nurses (*n* = 77)	PA (*n* = 4)	Overall (*n* = 101)	χ^2^, *p*-value
The organism responsible for EVD is					5.41, 0.247
Virus	18 (18.81)	61 (60.40)	4 (3.96)	84 (83.17)	
Bacterium	1 (0.99)	2 (1.98)	0	3 (2.97)	
Other	0	14 (13.86)	0	14 (13.86)	
Source of knowledge of EVD?					45.44, *p* > 0.001
During academic training	10 (9.90)	1 (0.99)	0	11 (10.89)	
From a colleague	0	13 (12.87)	0	13 (12.87)	
Media	8 (7.92)	60 (59.41)	4 (3.96)	72 (71.29)	
Workshop	2 (1.98)	2 (1.98)	0	4 (3.96)	
Other	0	1 (0.99)	0	1(0.99)	
Biosafety required for EVD processing					22.65, 0.001
BSL-1	0 (0)	2 (1.98)	0 (0)	2 (1.98)	
BSL-2	0 (0)	4 (3.96)	0 (0)	4 (3.96)	
BSL-3	11 (10.89)	8 (7.92)	0(0)	19 (18.81)	
BSL-4	9 (8.91)	63 (62.38)	4 (3.96)	76 (75.25)	
Biosafety required for EVD culture					20.93, 0.007
BSL-1	0 (0)	1 (0.99)	0 (0)	1 (0.99)	
BSL-2	0 (0)	2 (1.98)	0 (0)	2 (1.98)	
BSL-3	0 (0)	4 (3.96)	0 (0)	4 (3.96)	
BSL-4	11 (10.89)	8 (7.92)	1 (0.99)	20 (19.80)	
Don’t know	9 (8.91)	62 (61.39)	3 (2.97)	74 (73.27)	
Disinfectant required for attending to EVD patient					28.52, 0.001
70% Ethanol	4 (3.96)	1 (0.99)	0 (0)	5 (4.95)	
100% Ethanol	1 (0.99)	0 (0)	1 (0.99)	2 (1.98)	
0.5% Sodium Hypochlorite	2 (1.98)	6 (5.94)	1 (0.99)	9 (8.91)	
50% alcohol gel	1 (0.99)	4 (3.96)	0 (0)	5 (4.95)	
Other	10 (9.90)	50 (49.50)	2 (1.98)	62 (61.39)	
Don’t know	2 (1.98)	50 (49.50)	0 (0)	18 (17.82)	

*MDs* Medical Doctors, *PA* Physician Assistant

A total of 72 (71.29%) HCWs indicated media especially radio as the main source of information when asked where they first heard of EVD. This was significantly more than other sources (χ^2^ = 45.44, *p* < 0.05). When asked which biosafety level laboratory (BSL) is required to test sample from suspected patient with EVD, a total 19 (18.81%) indicated BSL-3 of which 11 (10.89%) were Medical Doctors, while 8 (7.92) and 1 (0.99%) were Nurses and Physician Assistants, respectively. A further 76 (75.25%), of which 9 (8.91%) were doctors, 62 (61.39%) Nurses and 3 (2.97%) Physician Assistants indicated BSL-4 (χ^2^ = 22.65, *p* = 0.001). A total 74 (73.27%) HCWs surveyed did not know which biosafety level laboratory is required to culture a sample from a patient suspected with EVD (χ^2^ = 20.93, *p* = 0.007).

When asked which disinfectant to use after attending to and caring for a suspected patient with EVD, only 8.91% (*n* = 9) could correctly identify bleach (0.5% Sodium Hypochlorite) which disinfectant to use (χ^2^ = 28.52, *p* = 0.001).

### Preparedness for an EVD outbreak by HCW category

Table 3 shows the levels of preparedness of HCWs to handle and manage EVD outbreak. When HCWs were asked if they considered their facilities sufficiently equipped to handle and manage EVD patients, 25.74% (*n* = 26) responded in the affirmative, while 54.46% (55) indicated otherwise. Of this, 14 (13.86%) were Medical Doctors, 39 (38.61%) Nurses and 2 (1.98%) were PA (χ^2^ = 2.66, *p* = 0.62). If they became accidentally infected with EVD after attending to a patient with EVD, 98 (97.03%) of those surveyed indicated they would accept to be isolated (χ^2^ = 4.69, *p* = 0.321). Meanwhile, 44.55% (*n* = 45) of HCWs would willingly attend to an EVD suspected patient (χ^2^ = 8.03, *p* = 0.09).

**Table 3:** Preparedness for an EVD outbreak by HCW category

Question	HCW category Total, (%)				
	MDs (*n* = 20)	Nurses (*n* = 77)	PA (*n* = 4)	Overall (*n* = 101)	χ^2^, *p*-value
Whether facility is equipped to handle EVD cases					2.66, 0.62
Yes	4 (3.96)	21 (20.79)	1 (0.99)	26 (25.74)	
No	14 (13.86)	39 (38.61)	2 (1.98)	55 (54.46)	
I don’t know	2 (1.98)	17 (16.83)	1 (0.99)	10 (19.80)	
Whether health staff agrees to be isolated or not					4.69, 0.321
Yes	19 (18.81)	75 (74.26)	4 (3.96)	98 (97.03)	
No	0 (0)	2 (1.98)	0 (0)	2 (1.98)	
Maybe	1 (0.99)	0 (0)	0 (0)	1 (0.99)	
Willingness to attend to an EVD patient					8.03, 0.09
Yes	9 (8.91)	36 (35.64)	0 (0)	45 (44.55)	
No	6 (5.94)	25 (24.75)	4 (3.96)	35 (34.65)	
Maybe	5 (4.95)	16 (15.84)	0 (0)	21 (20.79)	
Whether health staff is adequately trained or not					0.98, 0.614
Yes	1 (0.99)	8 (7.92)	0 (0)	9 (8.91)	
No	19 (18.81)	69 (68.32)	4 (3.96)	92 (91.09)	
Confidence level in handling an EVD suspected patient					13.09, 0.11
No confidence	3 (2.97)	15 (14.85)	1 (0.99)	19 (18.81)	
Little confidence	10 (9.90)	13 (12.87)	1 (0.99)	24 (23.76)	
Confident	3 (2.97)	29 (28.71)	1 (0.99)	33 (32.67)	
Very confident	3 (2.97)	15 (14.85)	0 (0.99)	18 (17.82)	
Extremely confident	1 (0.99)	5 (4.95)	1 (0.99)	7 (6.93)	

*MDs* Medical Doctors, *PA* Physician Assistant

A total of 92 (91.09%) HCWs surveyed indicated they were not adequately trained to handle an EVD suspected case. When asked to rate their competence in handling an EVD suspected patient, 18.81% (*n* = 19) indicated they had little confidence and competence, while 6.93% (*n* = 7) indicated they were extremely confident to handle a suspected case of EVD (χ^2^ = 13.09, *p* = 0.11).

### Others as indicated by HCWs

Beyond EVD, we asked our survey population to name other epidemic prone diseases. Of the total number of HCWs interviewed, 56.43% (57/101) mentioned epidemic diseases of bacteria origin such as tuberculosis and cholera. A further 33.70% (34/101) named diseases of viral origin such as SARS, Flu, HIV, Lassa fever and dengue, while 9.90% (10) mentioned others referring to malaria.

When asked the form of compensation HCWs thought would be appropriate when taking care of an Ebola suspected patient, responses given included personal insurance (32/101), family compensation in case of death (31/101), money (30/101) and awards (8/101) while others also suggested job promotion (7/101), and others (18/101).

Our survey population recommended the provision of logistics and training as two key issues in the way forward in adequately preparing HCWs towards any epidemic prone diseases.

## Discussion

Many issues surrounding the preparedness of HCWs have been extensively discussed globally especially in the aftermath of the Severe Acute Respiratory Syndrome Coronavirus (SARS-CoV) and the Middle East Respiratory Syndrome (MERS)-CoV epidemic. While it is on record that the recent EVD outbreak recorded very high mortality among HCWs, to the best of our knowledge, only few studies have addressed these issues in anticipation of an EVD outbreak particularly in countries not hit by the EVD epidemic and especially in sub Saharan Africa, such a study is almost non-existent. Our study therefore assessed how prepared HCWs are in the face of a possible EVD epidemic.

The results of this survey showed that more than half (54.46%) HCWs indicated that their facilities were not ready to handle EVD cases. Nearly 92% indicated they were not adequately trained to handle an EVD suspected case and it is not surprising that less than 50% indicated they would willingly attend to a suspected patient. Moreover, nearly a third of HCWs would also want insurance for themselves and their families in case they were infected with EVD.

These results are clearly indicative of how ill-prepared the HCWs surveyed are in the face a potentially life threatening epidemic prone diseases, such as EVD in Ghana. In this study, only 25.7% of HCWs said their facility was sufficiently equipped to handle an EVD outbreak. Such low ratings of the hospitals by majority of HCWs is a mark of lack of confidence in their facilities preparedness and this may actually indicate a real lack of preparedness and readiness of the hospitals to handle not only EVD cases but potentially other epidemic prone diseases. Alternatively, it could also mean that HCWs were probably unaware of preparatory work and retooling of their facilities to handle EVD outbreak situation.

Willingness to work during outbreaks and emergencies is deemed a sense of duty even in the face of risk. In this study, less than 50% of HCWs indicated their willingness to work in the event of an EVD outbreak. Additionally, over one third indicated various forms of compensation for themselves or families in case of death or while taking care of an EVD case. This implies that if HCWs are assured or guaranteed that they and or their families would be taken care of in case of death or while taking care of an EVD case, they will willingly work in the face of any emergency scenario. The assumption is that HCWs would willingly work in the face of an infectious diseases emergency and respond appropriately; however, there are evidences of HCWs avoiding this “sacred duty” in caring for patients and would leave patients vulnerable in times of crisis [11]. In order to prevent HCWs from being infected while obliged to work even in the face of personal risk as required by their codes of ethics and professionalism, it is imperative to ensure that appropriate conventional standards, guarantees and effective public health practices are met to enable HCWs respond to such outbreaks so that they are not infected and or affected despite the risks they might face and continue to face [12]. Thus, appropriate training of HCWs as indicated by those surveyed during the study, coupled with retooling of some health facilities preparation is very critical in ensuring that they are equipped with the needed knowledge and tools needed to work with in the face of any epidemic.

General knowledge of EVD is crucial to adequately respond to and care for patients. Nearly 17% of our study population could not identify that EVD as caused by a virus. Arguably, infection control measures would be difficult and problematic for such HCWs. Less than 10% could correctly identify 0.5% Sodium Hypochlorite as the best disinfectant out of the many options provided. This strongly contradicts a similar study in Conakry conducted during the peak of the epidemic where 68% of HCWs knew the correct concentration of disinfectant [5]. While not trying to compare these two scenarios, this information may be vital in the realization that knowledge of HCWs in infection prevention and control measures is critical in their line of duty.

This study showed that most HCWs first heard of EVD through the media especially radio. This establishes the crucial role media plays in informing the general populace in such disease outbreaks. In Ghana, there are over 350 media outlets (radio and television put together) and majority of households either own a radio, television or have access to internet. Notwithstanding the media pluralism, it is still incumbent upon health institutions and facilities to organize special training on any emerging infectious disease that occurs globally to update the knowledge of HCWs.

Isolation is a key public health measure to prevent the spread of infectious diseases. In this study, over 97% of HCW indicated their willingness to comply and accept to be isolated in case they became infected after attending to suspected EVD patient. However, a small proportion of HCWs surveyed stated that they would be very unhappy, and this could ultimately affect compliance. Isolation is one of the oldest methods of controlling communicable disease outbreaks for patients [13]. However, it is worthy of note that less that 50% said they would be willing to attend to an EVD suspected patient and we suspected that this could be related to fear of personal safety [14]. Emergency response from an epidemic prone disease from an exotic virulent virus or pathogen will naturally spark some level of fear and skepticism among any group of individuals especially when their knowledge about the dynamics of the disease outbreak is low. There are stories of HCWs who have avoided the responsibility of treating patients [15] and this was apparent in the HIV/AIDS and Severe Acute Respiratory Syndrome-Coronavirus (SARS-CoV) during the 1980s and 2003, respectively where the fear of contact with suspected and infected patients gripped some HCWs [16, 17]. In the long run, this fear would likely affect their confidence and commitment to professionalism.

The results of this study point to the fact that knowledge and the provision of tools such as personnel protective equipment (PPE) and other logistics alone is not good enough strategy. There might be the need to as well address issues related to myth, and culture as well as assurances of upkeep should one be infected. The general outlook one’s country’s devotion to their health staff might be a contributory factor in all of this and cannot be ignored. However, getting HCWs inspired and feel safe in caring for such highly infectious disease outbreaks is critical. During our study, HCWs indicated various forms of compensation to be paid to them should they be affected in the case of EVD attack.

## Conclusions

This study had some inherent limitations. This was an exploratory study and our sample size was limited. Therefore, while not trying to generalize the results, we are of the opinion that this may be a reflection of HCWs in general. Additionally, since our study focused mainly on two health facilities, we are again careful in extrapolating these to other to reflect other facilities. Moreover, since this has not been a real experience, and a questionnaire-based survey, responses may not accurately reflect real-life experiences in the event of an EVD epidemic. Despite these limitations, the need for training was strong among HCWs. The results further demonstrate the ill-preparedness of health facilities, and the large proportion of HCWs unwillingness to attend to a suspected case of EVD. This thus calls for concerted efforts of health institutions and facilities to fully equip and prepare HCWs with the requisite tools and knowledge and ensuring competency to handle any epidemic prone disease.

## Abbreviations

CDC: Centre for diseases control
EPDs: Epidemic prone diseases
EVD: Ebola virus disease
HCWs: Health care workers
MEPI: Medical education partnership initiative
MERS-CoV: Middle east respiratory syndrome coronavirus
OPD: Out Patient Department
PDAs: Personal digital assistant device
PPE: Personnel protective equipment
SARS-CoV: Severe acute respiratory syndrome coronavirus
WHO: World Health Organization
